# *Ruta graveolens*, but Not Rutin, Inhibits Survival, Migration, Invasion, and Vasculogenic Mimicry of Glioblastoma Cells

**DOI:** 10.3390/ijms252111789

**Published:** 2024-11-02

**Authors:** Iolanda Camerino, Paola Franco, Adriana Bajetto, Stefano Thellung, Tullio Florio, Maria Patrizia Stoppelli, Luca Colucci-D’Amato

**Affiliations:** 1 Department of Environmental, Biological and Pharmaceutical Sciences and Technologies, University of Campania “Luigi Vanvitelli”, 81100 Caserta, Italy; iolanda.camerino@unicampania.it; 2 Institute of Genetics and Biophysics “A. Buzzati Traverso” (IGB-ABT), National Research Council, 16149 Naples, Italy; paola.franco@igb.cnr.it (P.F.); mariapatrizia.stoppelli@unicamillus.org (M.P.S.); 3 Department of Internal Medicine (DIMI), University of Genova, 16132 Genova, Italy; adriana.bajetto@unige.it (A.B.); stefano.thellung@unige.it (S.T.); 4 IRCCS Ospedale Policlinico San Martino, 16132 Genova, Italy; 5 Departmental Faculty of Medicine and Surgery, Saint Camillus International University of Health Sciences, 00131 Rome, Italy; 6 InterUniversity Center for Research in Neurosciences (CIRN), 80131 Naples, Italy

**Keywords:** glioblastoma, vasculogenic mimicry, chemotherapy, natural compounds, cancer stem cells

## Abstract

Glioblastoma (GBM) is the most aggressive type of brain tumor, characterized by poor outcome and limited therapeutic options. During tumor progression, GBM may undergo the process of vasculogenic mimicry (VM), consisting of the formation of vascular-like structures which further promote tumor aggressiveness and malignancy. The resulting resistance to anti-angiogenetic therapies urges the identification of new compounds targeting VM. Extracts of natural plants may represent potential therapeutic tools. Among these, components of *Ruta graveolens* water extract (RGWE) display a wide range of biological activities. To test the effect of RGWE on human GBM and rat glioma cell line VM, tube formation on a gelled matrix was monitored. Quantitative assessment of VM formation shows the clear-cut inhibitory activity of RGWE. Unlike rutin, one of the most abundant extract components, the whole RGWE strongly reduced the migration and invasion of GBM tumor cells. Moreover, RGWE induced cell death of GBM patient-derived cancer stem cells and impaired VM at sub-lethal doses. Overall, our data reveal a marked RGWE-dependent inhibition of GBM cell survival, migration, invasion, and VM formation. Thus, the clear-cut ability of RGWE to counteract GBM malignancy deserves attention, holding the promise to bring natural products to clinical use, thus uncovering new therapeutic opportunities.

## 1. Introduction

Gliomas represent the most frequent type of cancer of the central nervous system (CNS). GBM belongs to this larger, heterogeneous group of brain tumors, which are believed to arise from glial cells or their precursors and can be divided into astrocytomas and oligodendrogliomas according to the cell type that gives rise to the tumor itself. Among these tumors, grade IV glioma, known as glioblastoma (GBM), is the most aggressive, characterized by uncontrolled cellular proliferation, marked invasiveness, diffuse infiltration, hypervascularization, necrosis, resistance to apoptosis, and genetic instability.

When the tumor develops directly, in its most aggressive form, it is called primary (or de novo) GBM. It arises from neural stem cell precursors. Differently, secondary GBM derives from the evolution of low-grade gliomas in which a series of further mutations is induced in addition to key mutations present at the level of the tumor stem cells. Primary GBMs are more likely to occur in elderly patients (mean age 62 years), mostly affecting males, while secondary GBMs arise from lower grade astrocytomas or oligodendrogliomas and have a higher incidence in younger (mean age 45 years) and female patients. Secondary GBMs usually have a lower degree of necrosis than the primary form, are usually associated with a better prognosis, and the typical location is the frontal lobe [1].

First-line treatment of GBM is based on surgical resection, followed by radiotherapy and chemotherapy. Temozolomide (TMZ), the gold standard cytotoxic drug for treating gliomas, is a DNA-alkylating agent largely used in anti-GBM chemotherapy [2]. Nevertheless, the prognosis is very poor, with a median survival ranging from 12 to 15 months [2] and an average 5-year survival rate of about 5% [3].

As a matter of fact, GBM frequently develops resistance to chemotherapy, thus impairing successful treatment of patients. In this respect, cancer stem cells (CSCs) identified in GBM are regarded to be widely responsible for the biological aggressiveness and therapeutic resistance [4,5].

Notably, differently from other high-grade solid cancers, malignant gliomas do not rely on intravascular or lymphatic metastasis to spread, but they frequently populate and migrate along existing brain structures, including nerve tracts, blood vessels, and the meninges. In particular, blood vessels are a critical substratum for GBM cell migration [6], which use perivascular space as a conduit for invasion [7].

Angiogenesis and vasculogenic mimicry (VM) are processes well known to ensure tumor blood supply for proliferation and migration of GBM cells. In particular, VM, a peculiar process shared by many solid tumors, consists of the endothelial-independent formation of functional vascular-like structures, nourishing tumor tissues by providing nutrient and oxygen supply [8]. This phenomenon likely results from the ability of tumor cells to transdifferentiate into endothelial-like cells, acquiring the capability to form tube-like structures secreting matrix proteins, such as collagens IV and VI, proteoglycans, heparan sulfate, and laminin, and supporting the formation of tubular networks within the tumor mass [9]. However, they are anatomically different from blood vessels, as they include a basal lamina behind the endothelial cells (ECs). Overall, VM is associated with poorly differentiated tumors and is related to a high degree of tumor infiltration and metastasis [10].

Although tubes generated via VM are negative for the endothelial marker CD31, they express Vascular Endothelial-cadherin (VE-cadherin), a key protein involved in cell interaction and in vasculogenesis [11]. Furthermore, in tumor biopsies, VM formation is detected by periodic acid–Schiff (PAS) staining combined with the absence of specific endothelial cell markers [12].

Being an endothelial-independent blood supply system, based on tumor cell plasticity, the formation of vascular-like structures in VM relies on a complex network of intracellular signaling such as the vascular endothelial growth factor (VEGF) pathway, detected in GBM tissue samples and glioma stem cells (GSCs), and related to self-renewal, tumorigenicity, and tubular formation [13].

To counteract GBM malignancy and overcome GBM resistance to current therapies, it is urgently needed to identify new and effective therapeutics, including natural products, the latter being a rich source of bioactive molecules that, over the years, have found application in the treatment of many diseases [14,15].

A number of compounds capable of inhibiting VM have been tested over the years. For example, histone deacetylase inhibitors (HDACis) have been revealed to be suitable anti-VM candidates [16]. Among them, suberoylanilide hydroxamic acid (SAHA, vorinostat) and trichostatin A (TSA) were first approved by Food and Drug Administration (FDA) for the treatment of several pathologies [17]. Nevertheless, a limited success resulted from the use of HDACis, as single agents against solid tumors in clinical trials, compared to the hematological malignancies [18]. However, in a previous work HDACis, when administered at sub-lethal doses, showed the ability to inhibit vascular-like structure formation [19]. It is worth noting that one of these compounds, TSA, which selectively inhibits class I and II HDACs, is a natural compound isolated from *Streptomyces hygroscopicus* bacteria, first studied for its antifungal activity [20].

A growing interest in therapies based on plant-derived drugs and/or on natural extracts encouraged the identification of novel compounds [21]. *Ruta graveolens* belongs to the rutaceae family, whose greatest distribution is found in the Mediterranean region. *Ruta* species have been employed for more than two millennia in traditional medicines and their potential benefits are still appreciated [22]. As a matter of fact, *Ruta graveolens* extracts are used for the treatment of different disorders, including ophthalmic diseases, rheumatisms, and dermatitis [23,24]. The water extract of this plant (RGWE) contains molecular species belonging to different classes of natural products, including alkaloids, coumarins, essential oils, and flavonoids [25]. Many of these are of great medical interest, as they exhibit a wide range of biological properties, like antioxidant, anti-inflammatory, and cytotoxic activities in human cancer cells as well as general anti-tumor activity [22].

We have previously shown that RGWE is cytotoxic for human GBM cell lines while leaving neuronal cells unharmed [26]. The current study was aimed at investigating whether sub-lethal concentrations of RGWE may counteract VM generated by GBM cells. For this purpose, we analyzed the ability of cancer cells to form tubes on a gelled matrix, as well as to migrate and invade, the two latter being prerequisites for vessel formation. Moreover, given the importance of GSCs in GBM resistance to therapy, we also evaluated the effects of different concentrations of RGWE on patient-derived GSC viability. Finally, here the ability of sub-lethal doses of RGWE to inhibit VM from a GSC culture was also investigated.

The findings that RGWE is able to affect GSC viability and to act as a strong inhibitor of both cell lines, as well as GSC-derived vascular-like structures, encourages further studies of RGWE as an anti-GBM adjuvant treatment.

## 2. Results

### 2.1. Glioblastoma Cells Form Vascular-like Structures in Extracellular Matrix

**Figure 1:**
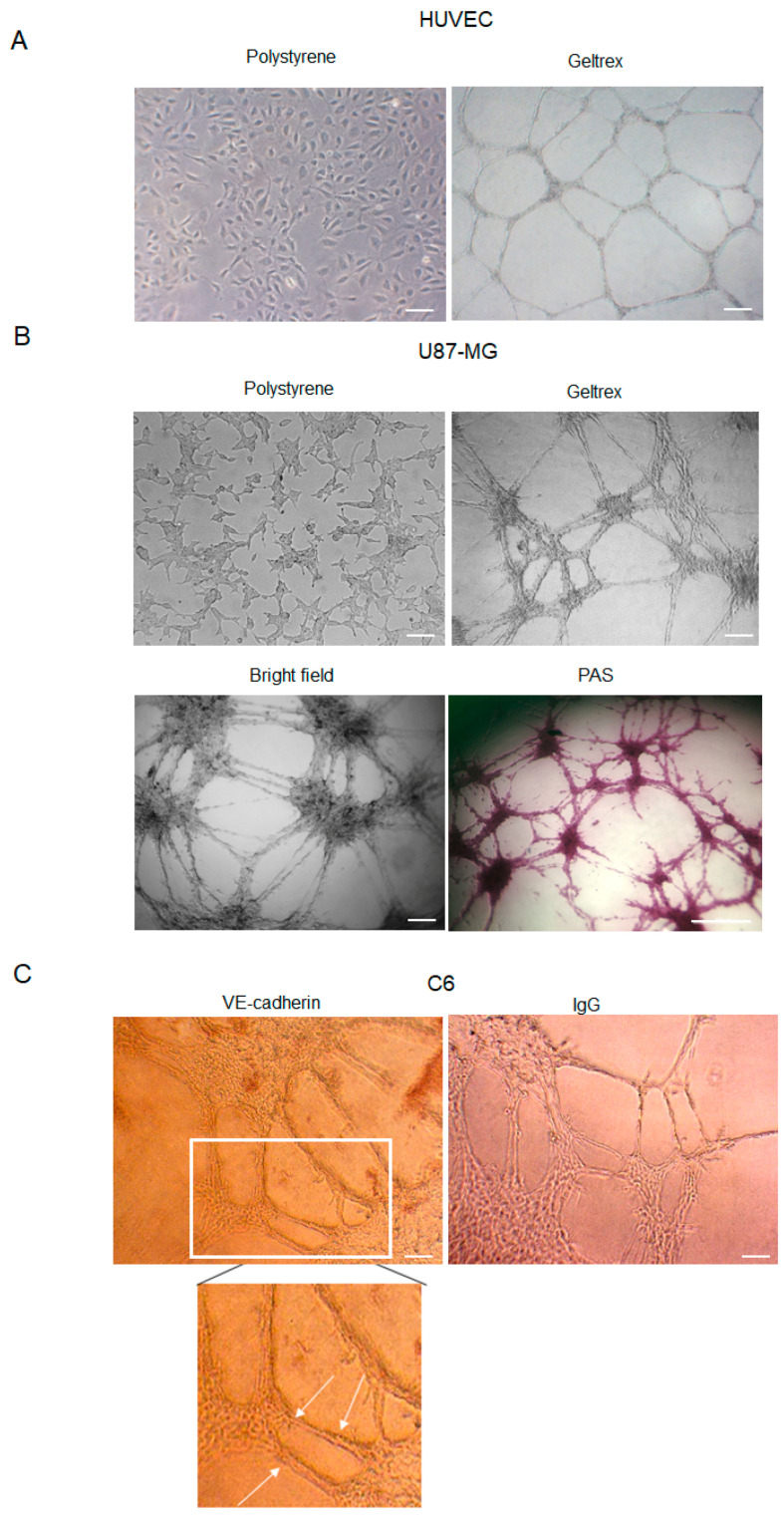
Tube formation by HUVEC, U87-MG, and C6 cells. (**A**) Representative images of HUVEC cells cultured in polystyrene dishes with or without ECM (Geltrex^TM^). Images were obtained by analyzing cells under the Inverted Microscope Axiovert 25 at 5× magnification, scale bars: 50 µm. (**B**) Representative images of U87-MG GBM cells cultured in polystyrene dishes, in a bright field or stained with PAS. Images were obtained by analyzing cells under the Inverted Microscope Axiovert 25 at 10× magnification in a bright field (scale bars: 100 µm) or under the Inverted Microscope DMI Leica 6000 at 10× magnification, for PAS staining, scale bar: 500 µm. (**C**) Immunocytochemistry assay on C6 glioma cells stained with anti-VE-cadherin antibodies. Antibody positivity (arrows) was revealed by DAB chromogenic substrate. Controls were incubated with secondary Ab (IgG) to exclude false positive signals. Images were obtained by observing cells under the Inverted Microscope Axiovert 25 at 10× magnification, scale bars: 100 µm.

HUVEC cell lines represent a suitable model to study angiogenesis in vitro. Furthermore, it is well established that several glioma cell lines are able to form vascular-like structures (Figure 1A) once plated onto a gelled extracellular matrix [27]. Here, we show that U87-MG cells assume an endothelial-like phenotype if seeded on Geltrex^TM^ (Thermofisher Scientific, Waltham, MA, USA) for 24 h, compared to cells cultured on polystyrene dishes (Figure 1B). Differentiating U87-MG cells progressively change morphology, elongate filamentous protrusions, and contact each other, ultimately forming vessel-like round structures. The time-dependent growing number of cell intersections or branching points may be evaluated to quantitate the phenomenon.

Because VM formation is inferred by the occurrence of PAS^+^ CD31^−^ vascular-like structures, Geltrex^TM^-seeded U87-MG cells were stained with PAS (Figure 1B). Moreover, consistently with previous results, our data confirmed that GBM cells form vascular-like structures positive for the VE-cadherin antibody (Figure 1C), a known marker of VM.

### 2.2. RGWE Inhibits U87-MG Vasculogenic Mimicry

**Figure 2:**
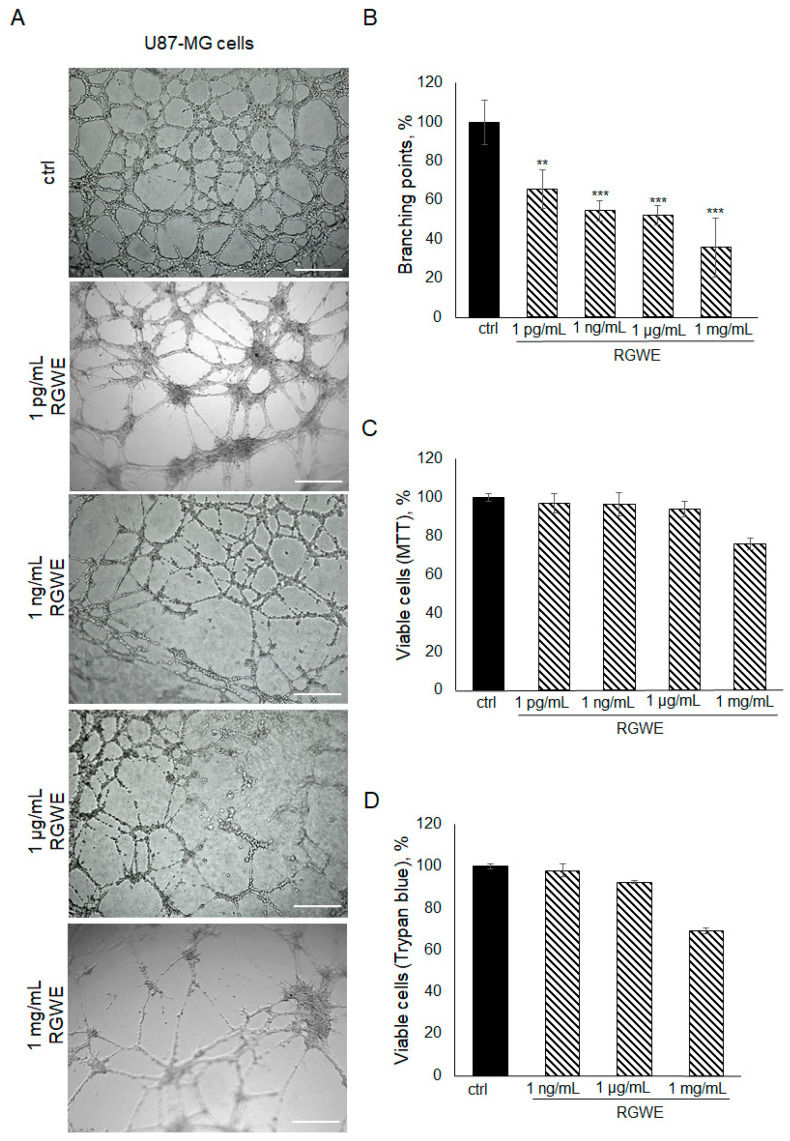
Effect of *Ruta graveolens* on U87-MG tube formation. (**A**) Representative images of VM formation of U87-MG cells in control (ctrl) and in treated samples at the specified concentrations. Branching point number was evaluated in each well by using the Inverted Microscope Leica DMI 6000 at 10× magnification, scale bars: 250 µm. (**B**) Quantification of branching points, defined as the intersection of at least three points [19]. They were counted in each well and expressed as a percentage of the ctrl, taken as 100%. The cell rate viability was tested by an MTT assay (**C**) and Trypan blue exclusion test (**D**). ** *p*-value < 0.005; *** *p*-value < 0.001.

To investigate the effects of RGWE on the ability of GBM cells to form tube-like structures in culture, U87-MG cells were plated on Geltrex^TM^ in the absence or presence of increasing concentrations of RGWE over a 24 h time-frame. As shown in Figure 2A,B, a reduction in branching point formation by 35% (±10), 46% (±5), 48% (±5), and 65% (±12) was observed in the presence of 1 pg/mL, 1 ng/mL, 1 µg/mL, and 1 mg/mL, respectively. Importantly, none of the concentrations tested affected the U87-MG cell viability rate (Figure 2C,D). In conclusion, RGWE remarkably inhibited the formation of vascular-like structures by U87-MG cells, without a significant interference with cell proliferation, which was minimally reduced only at the highest concentration tested.

### 2.3. RGWE Reduces U87-MG Cellular Migration and Invasion

**Figure 3:**
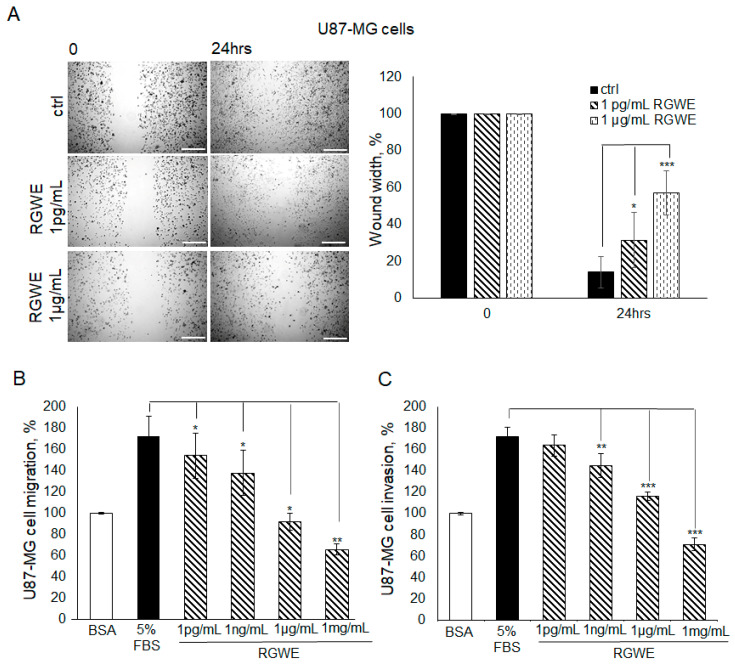
Effect of *Ruta graveolens* on U87-MG cellular migration and invasion in wound healing assays and chemotaxis assays. (**A**) Representative photograms of U87-MG cellular migration in wound healing assay in controls (ctrl) and in treated samples at the specified concentrations within 24 h. The images were obtained using the Inverted Microscope Leica DMI 6000 at 10× magnification, scale bars: 250 µm. Wound widths were quantitated by averaging the measurement of the wound margin distance at three points for each well. Results are expressed as wound width, as a percentage of the initial distance at T0, taken as 100%. (**B**) Directional migration assay in Boyden chambers of U87-MG exposed to 1 mg/mL Bovine serum albumin (BSA), 5% fetal bovine serum (FBS), or to the indicated concentrations of RGWE. (**C**) Directional invasion assay in Boyden chambers of U87-MG exposed to 1 mg/mL BSA, 5% FBS, or to the indicated concentrations of RGWE. Migrated and invaded cells were counted and expressed as a percentage of the cells recovered in the absence of chemoattractant (random migration/invasion, respectively), taken as 100% (BSA). * *p*-value < 0.05, ** *p*-value < 0.005, *** *p*-value < 0.001.

Initially, the VM process is characterized by a marked motility of cells contacting each other to form connections and round structures, suggesting that cell migratory ability is an important prerequisite to VM formation. Thus, the RGWE sensitivity of migrating and invading cells was tested in wound healing and Boyden chamber migration/invasion assays. In particular, Boyden chamber assays carried out using a filter precoated with collagen IV assess migration while those using filters further pre-coated with Matrigel evaluate invasion (see Section 4 for details). As shown in Figure 3A, in the wound healing assay, in the presence of serum, U87-MG cells reduced the distance between edges by 90% (±4.2) in 24 h. In contrast, in the presence of 1 pg/mL or 1 µg/mL RGWE, the percentage of wound closure inhibition was 33% (±9.4) and 62% (±6.1), respectively.

Furthermore, in Boyden chamber directional migration assays, in the presence of 5% FBS, U87-MG cells exhibited almost doubled basal migration, whereas an inhibition of 13% (±10.5), 20% (±10.5), 47% (±8), and 62% (±5) was observed in the presence of increasing concentrations of RGWE (Figure 3B).

RGWEs also exhibit a remarkable inhibitory ability in invasion assays, the percentages of inhibition being 16% (±11), 33% (±4), and 59% (±6), in the presence of 1 ng/mL, 1 µg/mL, and 1 mg/mL of RGWE, respectively. Overall, these data indicate that RGWE is able to strongly reduce the migration and invasion of U87-MG cells (Figure 3C).

### 2.4. The Effect of Rutin on U87-MG Ability to Migrate, Invade, and Form VM

**Figure 4:**
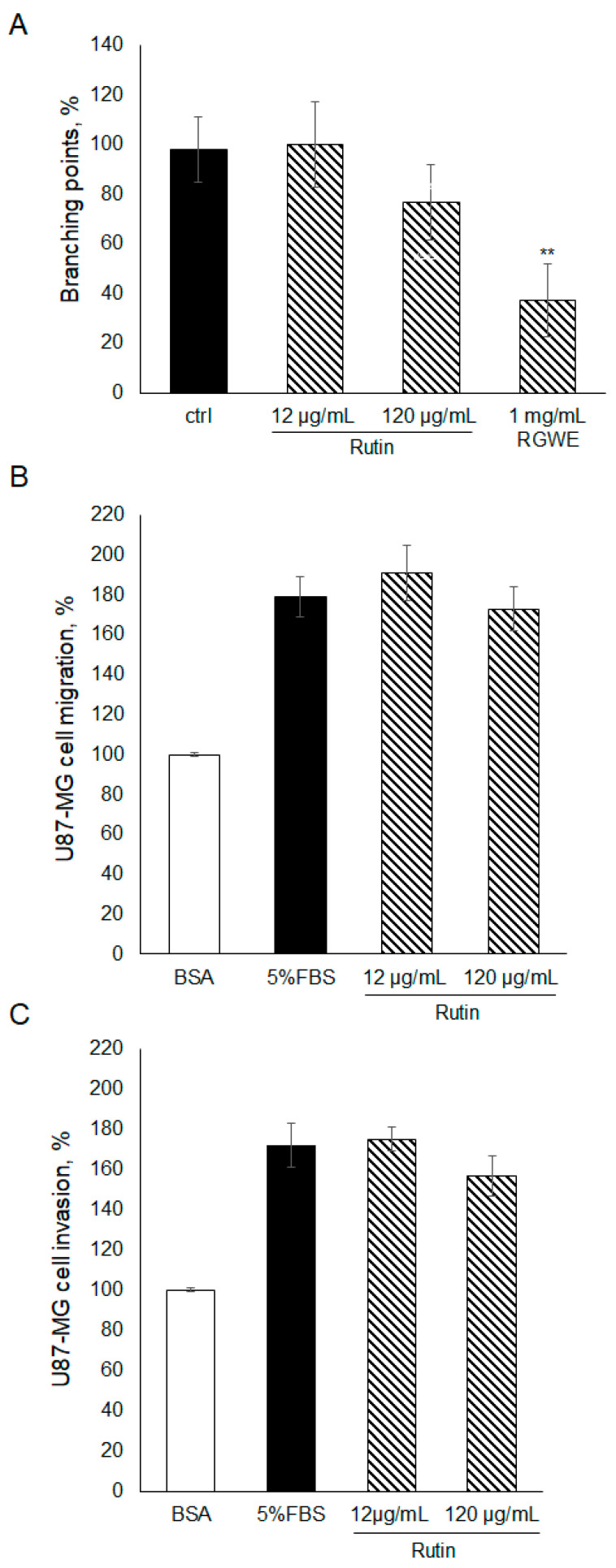
Effect of rutin on U87-MG VM, migration, and invasion. (**A**) U87-MG cells were plated on Geltrex^TM^, exposed to the indicated concentrations of rutin or to the whole extract, and branching points were counted as described in the legend to Figure 2. U87-MG cells were subjected to a migration assay (**B**) or to an invasion assay (**C**) in the presence of rutin at the specified concentrations, according to the procedure described in the legend to Figure 3. ** *p*-value < 0.005.

To gain insights into the components of RGWE producing the inhibitory effects on VM and migration of GBM cells, rutin, which is one of the major components of RGWE, was considered. In this set of experiments, rutin was tested at dosages occurring in the analyzed extracts of *Ruta graveolens* [26]. To this end, rutin was tested in VM, migration, and invasion assays, as shown in Figure 4. No statistically significant changes were observed in the presence of rutin, compared to untreated samples. Importantly, the activity of the rutin solution was tested, analyzing its scavenging properties [26]. Rutin was shown to exert anti-oxidative activity. Overall, the results indicate that rutin alone had little or no effect on U87-MG ability to migrate, invade, and form vascular structures, suggesting that other components may sustain the anti-tumoral activity of RGWE.

### 2.5. The Effect of RGWE on U251-MG GBM Cells

**Figure 5:**
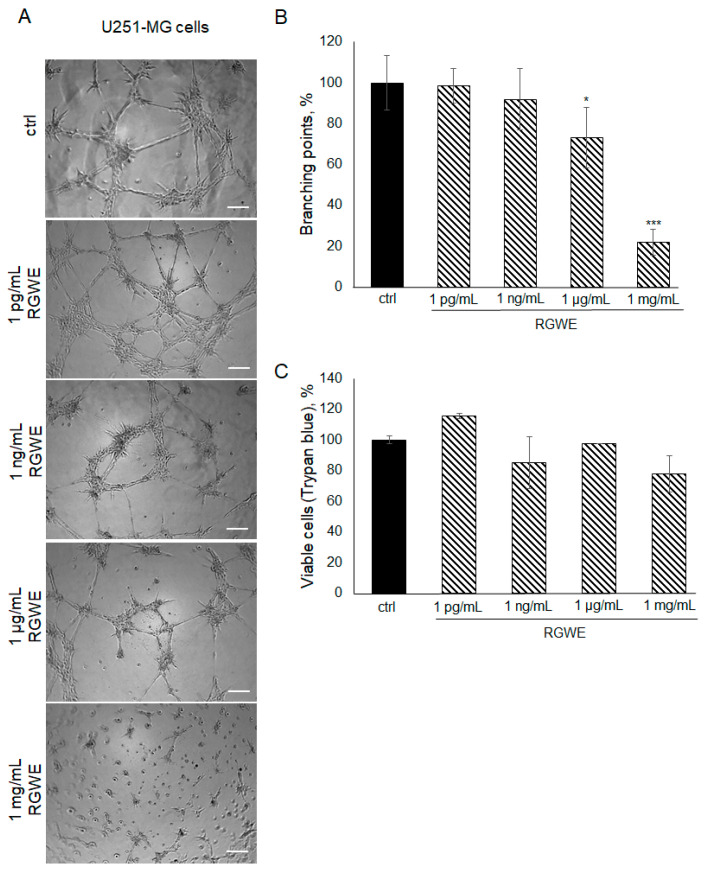
Effect of *Ruta graveolens* on U251-MG VM and viability. (**A**) Representative images of VM formation by U251-MG cells in control (ctrl) and in treated samples at the specified concentrations. Images were obtained analyzing cells under the Inverted Microscope Axiovert 25 at 5× magnification, scale bars: 50 µm. (**B**) Branching point numbers were evaluated as described in the legend to Figure 2. (**C**) Cell rate viability was tested by a Trypan blue assay. * *p*-value < 0.05; *** *p*-value < 0.001.

To investigate whether the inhibitory effects of RGWE are limited to the U87-MG cell line or might be extended to other GBM human cells, the U251-MG glioma cell line was also taken into consideration. Plated onto a gelled extracellular matrix, U251-MG cells undergo VM in a time-dependent manner (with a maximal effect in 24 h). In the presence of RGWEs (1 pg/mL, 1 ng/mL, 1 µg/mL, and 1 mg/mL), U251-MG cells experience a remarkable inhibition of VM, starting at the concentration of 1 µg/mL (Figure 5A,B). No significant changes were observed using 1 pg/mL and 1 ng/mL of RGWE, whereas a 27% (±14) and 79% (±6.42) reduction in branching point number was observed in the presence of 1 µg/mL and 1 mg/mL RGWE, respectively. This inhibitory effect is not due to a reduction in cell proliferation rate, as RGWE does not affect cell growth in a 24 h time-frame (Figure 5C).

Furthermore, the effects of RGWE were also tested in migration and invasion assays.

**Figure 6:**
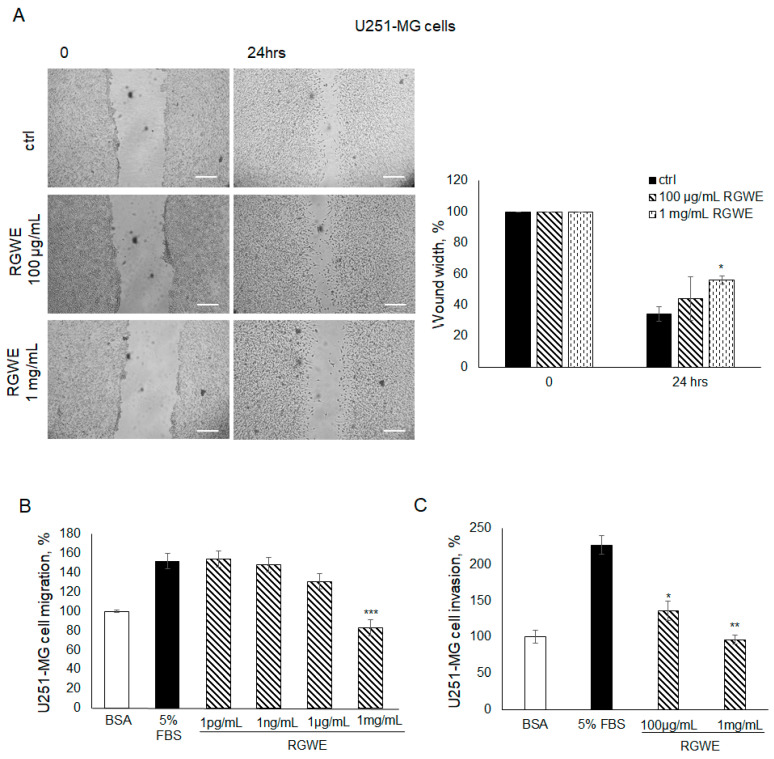
Effect of *Ruta graveolens* on U251-MG migration and invasion. (**A**) Representative photograms of U251-MG cellular migration in wound healing assay in controls (ctrl) and in treated samples at the specified concentrations within 24 h. The images were obtained using the Inverted Microscope Axiovert 25 at 5× magnification, scale bars: 50 µm. Wound widths were quantified as specified in the legend to Figure 3. (**B**) Analysis of U251-MG migration in Boyden chambers in the presence of the indicated concentrations of RGWEs, according to the procedure described in the legend to Figure 3. (**C**) Analysis of U251-MG invasion in Boyden chambers in the presence of the indicated concentrations of RGWE, according to the procedure described in the legend to Figure 3. * *p*-value < 0.05, ** *p*-value < 0.005, *** *p*-value < 0.001.

As shown in Figure 6A, in the wound healing assays, the cells migrate by closing the wound by 66% (±4.7) in 24 h. In contrast, in the presence of 100 µg/mL or 1 µg/mL of RGWE, the percentage of wound closure inhibition was 44% (±14) and 56% (±2.5), respectively.

In migration assays conducted in Boyden chambers, 5% FBS increased U251-MG cell basal migration by 1.5 times, whereas a 46% (±8.5) inhibition of migration was observed only in the presence of 1 mg/mL, which is the highest concentration of RGWE (Figure 6B). At lower concentrations, no significant difference was observed, unlike in the U87-MG cell line, which is sensitive to pico-nanograms of extract (Figure 3B). A similar observation applies to the concentrations of RGWE inhibiting VM, in the range of µg/mL-mg/mL, whereas U87-MG cells are inhibited already at 1 pg/mL (Figure 2B). Similarly to the migration assays, results obtained in invasion assays showed that a significant inhibition was observed using higher doses of RGWE. In particular, a 41% (±13) and a 58% (±6) inhibition of invasion was observed in the presence of 100 µg/mL and 1 mg/mL of RGWE, respectively (Figure 6C).

Overall, the data show that, although U251-MG cells are less sensitive than U87-MG to the anti-tumoral activity of RGWE, their ability to migrate, invade, and form VM are markedly inhibited.

### 2.6. The Effect of RGWE on C6 Cell Migration and VM

**Figure 7:**
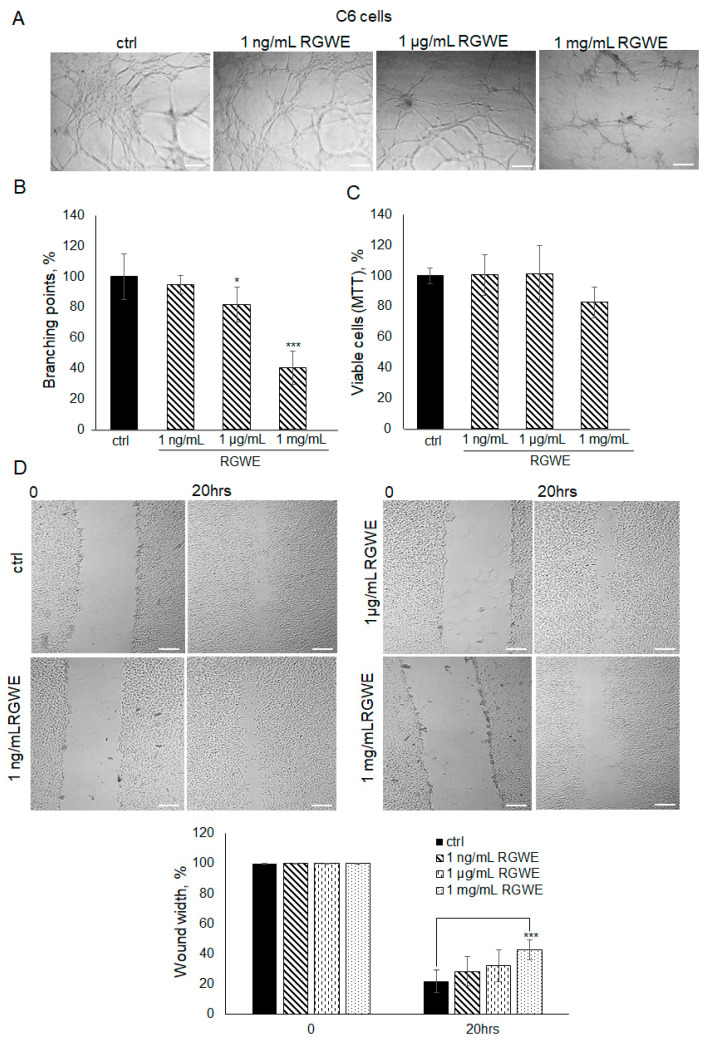
Effect of *Ruta graveolens* extract on C6 tube formation and wound healing closure. (**A**) Representative images of VM formation by C6 cells in control (ctrl) and in RGWE-treated samples at the specified concentrations. Branching point number was evaluated in each well by using the Inverted Microscope Axiovert 25 at 5× magnification, scale bars: 50 µm. (**B**) Quantification of branching points. (**C**) Cell rate viability of C6 exposed to the indicated concentrations of RGWEs for 24 h was tested by an MTT assay. (**D**) Representative photograms of C6 wound healing assay in control (ctrl) and in treated samples at the specified concentrations for 20 h. Images were analyzed under the Inverted Microscope Axiovert 25 at 5× magnification and wound widths were quantified as specified in the legend to Figure 3. Scale bars: 50 µm. * *p*-value < 0.05, *** *p*-value < 0.001.

It is known that C6 rat glioma cells are able to form vascular-like structures in 16 h [19]. In this experiment, C6 cells were seeded on Geltrex^TM^ and observed after 16 h. Unlike control cells, when C6 cells were seeded in the presence of 1 mg/mL RGWE, the number of branches was reduced by 60% (±10.63), whereas a scarce reduction was observed at 1 µg/mL (Figure 7A,B). Similarly to human GBM cell lines, 1 mg/mL of RGWE did not affect cellular proliferation (Figure 7C). In the wound healing assays, 1 mg/mL RGWE reduced wound width closure by 60% (±6.8), whereas lower concentrations were inefficacious (Figure 7D). Overall, the results indicate that, although C6 rat cells require higher concentrations of RGWE than human GBM cells, they do respond to the inhibition by RGWE in tube formation and in wound healing assays.

### 2.7. RGWE Is Able to Affect Survival and VM Potential of Patient-Derived GSCs

**Figure 8:**
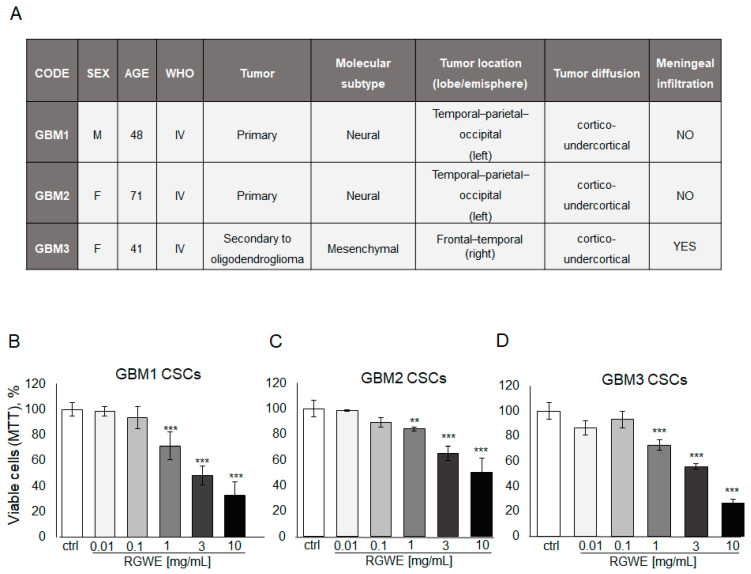
Effect of *Ruta graveolens* on patient-derived GBM GSC survival. (**A**) Table reporting clinical characteristics of patients and tumors selected for the isolation of CSCs. Cell rate of GBM1 (**B**), GBM2 (**C**), and GBM3 (**D**) primary culture viability by MTT assay in 72 h. ** *p*-value < 0.005; *** *p*-value < 0.001.

Like other tumor types, GBM tumors in vivo harbor a population of cancer stem cells with unlimited proliferative potential, being required for tumor development and maintenance [28]. To investigate the activity of RGWE on primary GSCs, specimens derived from three GBM patients were employed to isolate cancer stem cells and obtain GSC-enriched cultures. The patients’ tumor characteristics are reported in Figure 8A.

First, cell survival of cultured GSCs, exposed to increasing concentrations of RGWE (0.01–10 mg/mL) for 72 h, was tested by MTT assay. A dose-dependent inhibition of cell survival, starting at 1 mg/mL, without significant differences among GBM1, GBM2, and GBM3 cultures, was detected (Figure 8B,C,D, respectively). Remarkably, all three GSC cultures display responsiveness to the inhibition by RWGE extract within the same concentration range (1–10 mg/mL) (Figure 8B).

**Figure 9:**
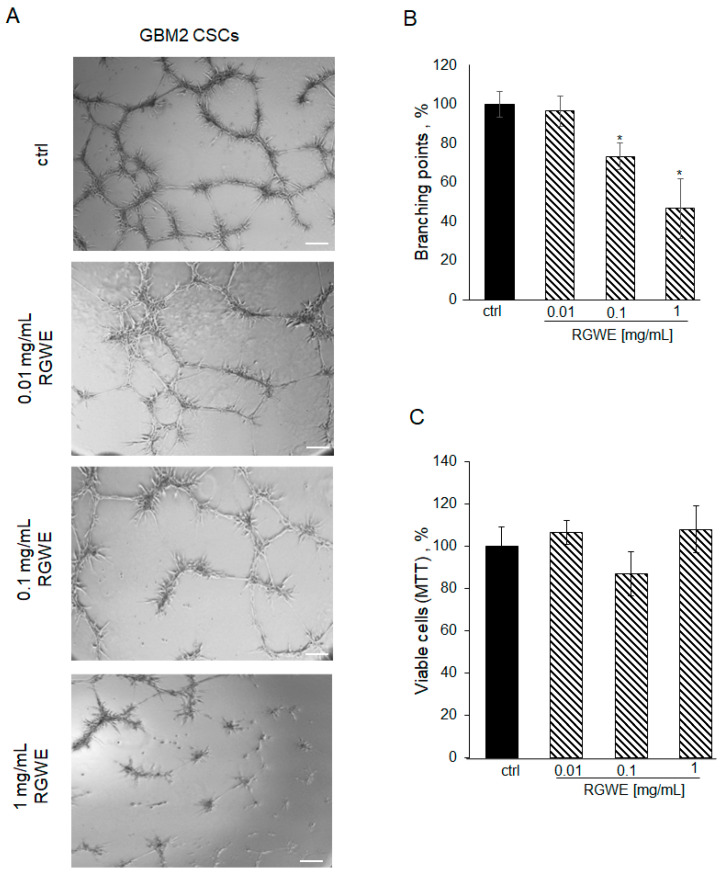
Effect of *Ruta graveolens* on tube formation by patient-derived GBM CSCs. (**A**) Representative images of VM formation by GBM2 GSCs in control (ctrl) and in treated samples at the specified concentrations. Images were obtained by analyzing cells under the Inverted Microscope Axiovert 25 at 5× magnification, scale bars: 50 µm. (**B**) Quantification of branching points was performed as described in Figure 2 legend. (**C**) Cell rate viability tested by MTT assays at 24 h. * *p*-value < 0.05.

However, RGWE required 72 h to reduce GSC viability in the cultures derived from all three GBMs. Thus, we tested the efficacy of RGWE in a VM assay also in these cultures. First of all, patient-derived GSCs seeded on a GeltrexTM matrix, under the same conditions described for the GBM cell lines, develop vascular-like structures in 24 h (Figure 9A, ctrl). The addition of RGWEs at 0.1 and 1 mg/mL caused a 25% (±6.7) and 50% (±15.3) reduction in branching number, respectively (Figure 9B).

To investigate whether this reduction was due to the inhibition of cell viability in the 24 h time-frame required by the VM assay, an MTT assay on proliferating GSCs was performed. As shown in Figure 9C, GSC proliferation is not affected by exposure to RGWE for 24 h. Overall, the data show that VM is inhibited by RGWE in 24 h without affecting cell proliferation, whereas prolonged exposure to the plant extracts for 72 h leads to inhibition of GSCs proliferation at 1–10 mg/mL RGWE concentrations.

## 3. Discussion

GBM is a deadly type of cancer whose prognosis is extremely poor despite the combined therapy involving debulking surgery, radiotherapy, and chemotherapy. Although TMZ is the drug of choice, since its approval the overall median survival has only increased to 14.6 months compared to the 12.1 month median survival observed when radiation alone is administered. In addition, GBM patients treated with TMZ are likely to develop resistance and tumor recurrence occurs [29].

Moreover, the extreme ability of GBM to migrate and invade the surrounding tissues makes it difficult to complete surgical resection without affecting the brain’s functions [30]. Thus, new compounds and/or therapeutic approaches are urgently needed.

Among the well-known biological and pathological features of GBM, hypervascularization, together with the ability of GBM cells to migrate and invade brain tissues, are recognized mandatory GBM targets, representing important factors of worse prognosis [31].

For these reasons, bevacizumab, a humanized monoclonal antibody binding and blocking VEGF, a main angiogenic factor, has been introduced in anti-GBM therapy [32]. Unfortunately, in the large majority of cases, the clinical improvements are transient since the tumors restart growing and the patients will ultimately die [33]. Noteworthily, patients with bevacizumab-resistant GBMs exhibit a propensity towards perivascular invasion [34,35].

Among the factors held responsible for the resistance to anti-angiogenic therapy (i.e., bevacizumab), a central role is played by endothelial-independent vascular formation or VM [9]. VM is one of the different neovascularization mechanisms, consisting of the formation of vessel-like structures originated by cancer cells [36].

In the framework of the search for new anti-GBM compounds, we report the activity of RGWE, the aqueous extract of a natural plant. When administered at sub-lethal doses, RGWE is able to inhibit the formation of vascular-like structures by two human GBM cell lines U87-MG and U251-MG as well as a C6 rat glioma cell line in culture. Notably, the inhibition of VM at the RGWE maximum dosage (1 mg/mL) was approximately 60% for U87-MG and C6 cells and nearly 80% for U251 cells, suggesting a similar overall sensitivity for all three cell lines. These experiments also show that the inhibitory effect of RGWE does not show species specificity, since both human and rat cells are equally sensitive to 1 mg/mL RGWE. Regarding the minimum effective dose of RGWE, it has to be noted that U87-MG cells were the most sensitive to the action of RGWE, being inhibited at a concentration as low as 1 pg/mL (35%) but increasing the sub-lethal doses (i.e., 1 ng/mL, 1 µg/mL, and 1 mg/mL) led to higher inhibition of vessel-like formations by 46%, 48%, and 65%, respectively. As for U251-MG and C6 cells, we observed inhibition starting from 1 µg/mL with a decrease in tube formation of about 27% and 19%, respectively. The reason for the different sensitivity at low dosages of RGWE may be related to the underlying genetic landscape of each cell line, possibly interacting with different components of the natural extract.

On the other hand, it is worth noting that GBM cell lines exhibit a different sensitivity also following exposure to HDAC inhibitors (HDACi), as inhibitors of VM in vitro [19]. In our previous work, U87-MG cells were inhibited by trichostatin (TSA), entinostat (MS275), vorinostat (SAHA), and MC1568, whereas C6 cells were not responsive to SAHA and MC1568. Thus, tumors harboring different mutations are likely to display a selective sensitivity towards these molecules or alternatively, at least in this case, HDACi may discriminate among different species (i.e., human vs. rat).

The inhibitory activity of RGWE can be extended to human endothelial cells, as it hinders in vitro vessel network formation in a dose-dependent manner, by down-regulating VEGF-A and nestin gene expression via the MEK-ERK1/2 pathway, without affecting cell viability [26]. Notably, this pathway is well known to play an important role in vivo in angiogenesis, including cancer neo-angiogenesis [37]. In addition, it has been shown that VEGF-A silencing is overcome by the induction of VM that, in turn, fosters GSCs enrichment [38]. Overall, RGWE is able to inhibit vessel-like structures originated, in vitro, from both human endothelial as well as from human and rat GBM cells (U87-MG, U251-MG, and C6 cells), and therefore it may represent a potential useful tool to hinder neo-angiogenesis and VM, characterizing aggressive GBM.

To study VM in GBM cells, we conducted a tube formation assay on a gelled matrix. This is an in vitro assay widely used to study both normal and alternative angiogenesis such as VM by evaluating, in a quantitative manner, the cellular ability to form vessel-like structures. Although it is a simplistic model since it does not consider the other cell types of the tumor microenvironment, it is highly reproducible, widely used to identify compounds affecting vessel formation, and better able to dissect signal transduction than in vivo models including in GBM [19,39,40,41].

Furthermore, here we report that RGWE also strongly inhibits migration and invasion of the two human and the rat GBM cell lines. Migration and invasion are two important hallmarks of GBM, exerting a fundamental role in its pathogenesis and aggressiveness. Moreover, they are also prerequisites for vessel formation, including VM [39,42]. Consistent with tube formation assays, U87-MG cells proved to be sensitive to lower concentrations of RGWE than U251-MG and C6 cells (i.e., 1 pg/mL–1 ng/mL vs. 1 µg/mL–mg/mL, respectively).

It is noteworthy that the cell lines analyzed are among the most used in preclinical studies to test the potential therapeutic effects of a huge number of compounds on GBM. In addition, they have been instrumental in dissecting the signaling pathways occurring in this type of cancer. They share many key molecular features as well as some important biological characteristics. Nevertheless, they also display some notable differences at both the molecular and functional level [43]. Thus, experiments carried out in different cell lines rule out the possibility that the results obtained are due to the specific genomic cellular landscape of a single cell line. On the whole, it is not surprising that although all cell lines respond to RGWE, the magnitude of response is not same and the effects occur at different time-points. A mechanistic explanation of these differences will require a deep knowledge of the active principle/s involved and can be accomplished in a future work.

Concerning the potential therapeutic effect of sub-lethal concentrations of RGWE, it is worth mentioning that, recently, it has been shown that sub-lethal doses of doxorubicin, a widely used chemotherapeutic drug, foster the migration and invasion of human non-invasive breast cancer cells, raising the issue of the risk of pro-metastatic effects [44]. Indeed, a sublethal dose of cisplatin was also found to increase the migration and invasion of cancer cells [45]. As a matter of fact, from a clinical point of view, drug concentration at the tumor site may decrease over time due to an enlargement of the tumor mass and/or a reduction in blood perfusion. Therefore, sub-lethal concentrations of chemotherapeutic drugs are likely to occur in patients undergoing therapy. Hence, cancer drugs, besides being ineffective because of drug resistance, might possibly promote migration and invasion and worsen the clinical outcome of patients. Moreover, sub-lethal ionizing irradiation also promotes the migration and invasion of glioma cells in both in vitro and in vivo models [46,47]. Such a condition is likely to occur at the border of the tumor, in particular in the case of brachytherapy, and may explain the recurrence and the higher aggressiveness often observed following therapy [48].

Although unraveling the molecular mechanism underlying the above-mentioned effects of RGWE are beyond the aim of the present work, preliminary data show that in GBM cells, RGWE is able to downregulate the expression of VEGF mRNA in a dose-dependent manner (Supplementary Figure S1). It is worth noting that VEGF has been involved not only in angiogenesis but also in cancer cell migration and invasion as well as in VM of some types of tumors [49,50].

Rutin is a flavonoid glycoside also known as vitamin P, largely present in vegetables and fruits as well as in medicinal herbs such as *Ruta graveolens*. An LC ESI-Q/TOF MS analysis showed that rutin represents the most abundant compounds within RGWE [26]. Natural compounds have been a fundamental source of drugs currently used in cancer therapy. For example, vincristine and vinblastine from *Catharanthus roseus* as well as paclitaxel isolated from *Taxus brevifolia* are now used as cytotoxic agents for several cancer types [51]. Interestingly, non-toxic natural compounds have also been proven to exert a therapeutic role in cancer. For example, curcumin, derived from the rhizome of the plant *Curcuma longa*, was found to hinder the proliferation, migration, and invasion of GBM cells by interfering with the JAK/STAT3 signaling pathway [52]. Here, we found that rutin was unable to elicit any measurable effect on migration, invasion, and vessel-like formation in U87-MG GBM cells, the more sensitive among the tested cell lines. Hence, we assume that either rutin is not necessary or, alternatively, it is needed but in combination with other compounds present in the extract. This issue will require further investigation aimed at identifying the inhibitory components of RGWE by means of “omic” techniques for high-throughput screening [53,54,55,56,57].

However, the lack of efficacy of a single compound such as rutin is not surprising since, as in other cases, the overall effect observed (i.e., migration, invasion, and tube formation inhibition) is likely due to a complex interplay between a number of molecules present in different concentrations in the whole extract [58]. However, it is worth noting that, under our experimental conditions, rutin, but not RGWE, did not exert any effect either on GBM cell death or on vessel formation in HUVEC human endothelial cells [26,59]. On the contrary, rutin was able to ameliorate the brain damage in a rat model of transient focal brain ischemia, although a more potent neuroprotective effect was observed when RGWE was administered [60]. These findings suggest that also in this experimental setting, a combination of compounds elicited a more powerful effect.

Importantly, all the cell lines involved in our study have been shown to contain a subpopulation of GBM cancer stem cells [61,62]. Importantly, in this work, RGWE effects were also tested on GSCs isolated from three patients with GBM, including primary and secondary GBMs. It is widely accepted that GSCs are responsible for the frequent recurrence and extremely poor prognosis, despite multimodal therapy [63,64,65]. Here, we show that RGWE is able to decrease the viability of GSCs derived from all three patients, although showing a concentration–response curve shifted to the right. This lower RGWE potency in GSCs as compared to cell lines is somewhat expected since it is nowadays widely accepted that GSCs, but not bulk tumor cells, are able to self-renew and to form a heterogenous population of cancer cells characterized by overexpression of drug extrusion pumps and DNA repair mechanisms, which confer resistance to classic chemotherapic drugs as well as to radiotherapy [66]. Importantly, one such patient-derived GSC culture was also challenged with unlethal doses of RGWE, showing a significant decrease in vessel-like structure formation. Notably, GSCs are involved in the process of VM by means of mechanisms that are not fully understood, but likely related to trans-differentiation [67]. It is worth mentioning that, within a tumor mass, CSCs may be present in a quiescent state; thus, chemotherapy based on classic anti-proliferative drugs is ineffective [68].

In conclusion, the main effects exerted by RGWE on the GBM cells, here reported, are the following: (i) an antiproliferative effect on patient-derived GSCs when administered at lethal doses; (ii) decreased migration and invasion activity of human GBM cells and of a rat cell line with nonlethal doses; and (iii) a decreased vessel-like structure formation of human GBM cells, including a patient-derived GSC culture, as well as of a rat glioma cell line.

Previous work has shown that lethal doses of RGWE in GBM cell lines were nonharmful to neuronal differentiated cells, while TMZ or cisplatin was toxic [30]. Although this issue deserves further investigation, under our experimental conditions the effects of RGWE seem to be selective towards GBM cells.

Taken together, these findings indicate that RGWE is a promising tool as it is and/or to search for novel compounds useful to treat GBM. Further investigations are warranted to evaluate the translational feasibility of the current findings.

## 4. Materials and Methods

### 4.1. Cell Line Cultures

The human GBM cell lines, U87-MG and U251-MG, and rat glioma cell line, C6, were cultured in Dulbecco’s Modified Eagle Medium (DMEM) (Life Technologies, Paisley, UK) containing 10% fetal bovine serum (FBS) (Life Technologies, Paisley, UK), supplemented with 100 U/mL of Na–penicillin and 100 µg/mL of streptomycin sulfate. Human umbilical vein endothelial cells (HUVECs) were maintained in endothelial cell growth medium: EBM2 medium (Lonza Srl, Milano, Italy) supplemented with 1 µg/mL hydrocortisone and 1 ng/mL epidermal growth factor (EGF) and 10% FBS. Cells were cultured in a humidified tissue culture incubator at 37 °C, 5% CO_2_, and 95% air atmosphere. U87-MG cells were obtained from the ECACC (European Collection of Authenticated Cell Cultures Porton Down, Salisbury, UK), while U251-MG, C6, and HUVEC cells derived from ATCC (American Type Culture Collection, Rockville, MD, USA).

### 4.2. Human GBM Specimens and CSC Cultures

Three GBM specimens were obtained from the Neurosurgery Department of IRCCS Ospedale Policlinico San Martino (Genova, Italy), after obtaining patients’ written informed consent and institutional ethical approval by CER Liguria (ethic code 360/2019). Patients and tumor characteristics are reported in Figure 7A. Primary cell cultures were obtained and characterized as previously reported [69] and maintained in vitro in serum-free medium containing 1:1 DMEM-F12/Neurobasal™ (EuroClone, Milano, Italy/Gibco-ThermoFisher Scientific, Monza, Italy), B27™ supplement (Gibco-ThermoFisher Scientific), 2 mM L-glutamine (EuroClone), 1% penicillin–streptomycin (EuroClone), 15 μg/mL insulin (Sigma-Aldrich, Milano, Italy), 2 μg/mL heparin (Sigma-Aldrich), and completed with recombinant human bFGF (10 ng/mL; Miltenyi Biotec, Bologna, Italy) and EGF (20 ng/mL; Miltenyi Biotec). These culture conditions allow the formation of floating tumorspheres within 10–14 days, as an indirect index of self-renewal, and resulting enrichment of CSC-like cells [70]. To obtain more reproducible experimental conditions, cells were grown as monolayers in flasks coated with growth factor-reduced Matrigel™ (Corning, ThermoFisher Scientific, Milano, Italy), which ensure the maintenance of cancer stem cell features [71]. Tumor-initiating activity was confirmed by orthotopic xenografts in 6–8-week-old non-obese diabetic severe combined immunodeficient (NOD/SCID) mice, injecting 1 × 10^4^ cells [71,72].

### 4.3. Extract Preparation and Cell Treatments

*Ruta graveolens* is a plant of the Rutaceae family, very common in Italy, growing spontaneously below 1000 m above sea level. It is not a protected species and commonly cultivated, even conserved at the Experimental Section of Medicinal Plants at the Botanical Garden of Naples, Italy. The extract was obtained as previously described in [58]. Briefly, before the flowering stages, 250 g of leaves was harvested, chopped, and then boiled in 1 L of distilled water at 110 °C for 1 h. The extract was subsequently filtered through 0.22 μm filters (Millipore, Bedford, MA, USA), frozen under liquid nitrogen, and then lyophilized (VirTis-SP Scientific, ATS Life Sciences, Warminster, PA). Rutin, one of the major components, was used at concentrations of 12 and 120 µg/mL, corresponding to the doses present in 100 µg/mL and 1 mg/mL of *Ruta graveolens* water extract (RGWE), according to spectrophotometer studies [26]. Rutin was purchased from Sigma. When necessary for the experiments, RGWE and rutin were diluted with water to the standard concentration of 100 mg/mL and stored at −20 °C until further use. Further dilutions were conducted in DMEM at appropriate concentrations.

### 4.4. In Vitro Tube Formation Assay

The tube formation assay was performed in 96-well tissue culture plates in the presence of 50 µL/well of Geltrex^TM^ (Thermofisher Scientific, Waltham, MA, USA). The commercialized extracellular matrix was left to polymerize for 30 min at 37 °C. U87-MG, U251-MG, C6, and HUVEC cells were dissociated by trypsinization, washed in PBS (+Mg^2+^, +Ca^2+^), re-suspended in serum-free medium at 3 × 10^4^, 2 × 10^4^, 1 × 10^4^, and 2 × 10^4^ cells/100 µL well, respectively, plated onto Geltrex^TM^-stratified wells, and then monitored at 37 °C for 24 h, in the case of human glioma cell lines, and for 16 h, in the case of the rat glioma cell line. The evaluation of U87-MG branching points was performed using the Inverted Microscope DMI Leica 6000 (Zeiss, Milano, Italy) at 5–10× magnification, equipped with a Digital Camera DFC420, whereas U251-MG and C6 cells were analyzed with the Inverted Microscope Axiovert 25 (Zeiss) at 5–10× magnification, equipped with Zen 3.3 Software. The tube formation assay for the patient-derived GBM2 specimen was performed in µ-Slide Angiogenesis (Ibidi, Munich, Germany). Briefly, wells were coated with Matrigel^®^ (Sigma-Aldrich) and allowed to polymerize for 30 min at 37 °C. An amount of 10^4^ cells were re-suspended in serum-free medium, subsequently seeded on Matrigel^®®^, and then incubated for 24 h. Following incubation, each well was analyzed directly under the Inverted Phase Contrast Microscope Axiovert 25 (Zeiss). At the end of each experiment, the number of “branching points” was counted in 4/5 random fields using the ImageJ Software, version 1.8.0. The mean value ± standard deviation for each sample was reported [73].

### 4.5. PAS Staining

Three-dimensional tubes were fixed in 1.5% paraformaldehyde in Phosphate-Buffered Solution (PBS) 1× for 30 min at room temperature (RT). Afterward, quick washing with 1× PBS was performed, and wells were then incubated with 0.5% periodic acid for 10 min, washed with distilled water for 10 min, incubated with Schiff reagent for 25 min, and washed for 15 min with distilled water for microscope observation.

### 4.6. Immunocytochemistry

GBM cells were seeded onto Geltrex^TM^, as previously described. After 24 h, branching points were formed and tubes were fixed with 1.5% PFA solution for 20 min at RT. Afterwards, a blocking solution (PBS/10% FBS) was added to fixed tubes for 1 h at RT. Monoclonal antibody anti VE-cadherin (Sony Biotechnology Inc., San Jose, CA, USA) (1:50 in PBS) was left to act for 2 h at RT. Wells were then incubated with anti-mouse IgG HRP conjugated secondary antibody (ImmunoReagents Inc., Raleigh, NC, USA) (1:500 in PBS) for 1 h at RT. Negative controls were incubated with anti-mouse IgG only. A DAB substrate Kit (Vector Laboratories, Burlingame, CA, USA) was used to reveal antibodies, observing cells under the Inverted Microscope Axiovert 25 at 10× magnification.

### 4.7. Wound-Healing Assay

Random migration of U87-MG, U251-MG, and rat C6 cells was analyzed by a wound healing or scratch test assay. Briefly, U87-MG cellular migration was analyzed in 24-multiwell plates containing culture inserts for living analysis (Ibidi, Gmbh, Martinsried, Germany). An amount of 6 × 10^3^ U87-MG/insert/sample were plated in the presence of DMEM/10% FBS for 24 h. After removing Ibidi inserts, a rectangular space was formed, which could be invaded by surrounding cells. Images were captured at the indicated time-points using the Inverted Microscope Leica DMI 6000 at 10× magnification and analyzed with the Leica Application Suite Software, version 2.7.3 (Leica Microsystems, Milano, Italy). In the case of C6 and U251-MG, 10 × 10^4^ cells/well/sample and 12 × 10^4^ cells/well/sample, respectively, were plated in the presence of DMEM/10% FBS for 24 h; afterwards, the medium was removed, cells were washed with PBS, a scratch was made on the cellular monolayer with a pipette tip, and cells were then resuspended in DMEM/2.5%FBS and monitored for 20 h and 24 h, respectively. The images were acquired using the Inverted Microscope Axiovert 25 at 5× magnification, equipped with Zen 3.3 Software. The extent of wound closure is expressed as the average decrease in wound distance at three points, considering as 100% the distance between the monolayer margins at the time of wound formation.

### 4.8. Boyden Chamber Migration and Invasion Assays

Boyden chamber assays were performed using 8 µm pore size PVDF-free filters coated with collagen type IV (50 µg/mL, Sigma), as previously described [74]. Briefly, 1.1 × 10^5^ U87-MG and U251-MG cells were pre-treated for 40 min at 37 °C with RGWE (1 pg/mL, 1 ng/mL, 1 μg/mL, and 1 mg/mL) or rutin (12 μg/mL and 120 μg/mL). The cells were then allowed to migrate for 3 h at 37 °C toward DMEM/5% FBS. For invasion assays, filters were further coated with Matrigel (50 µg/mL, Sigma) and U87-MG and U251-MG cells were pre-treated as reported for the migration assay and invaded for 5 h at 37 °C by DMEM/5% FBS. At the end of both assays, cells on the lower filter surface were fixed, stained with hematoxylin, and then counted under the Optical Microscope Axiophot (Zeiss) at 20× magnification. The results were reported as a percentage of the basal random cell migration or invasion in the absence of chemoattractant taken as 100%.

### 4.9. Trypan Blue and MTT Viability Assays

An amount of 5 × 10^4^ U87-MG cells/well in 12-multiwell dishes and 5 × 10^3^ U251-MG cells/well in 24-multiwell dishes were grown in DMEM/10% FBS for 24 h. Samples were treated with RGWE and rutin at the indicated concentrations for 24 h, and then trypsinized and stained with 0.2% Trypan blue (1:1 with cell suspension). The total and the viable cell numbers were counted, and the number of viable cells was reported as a % of the control.

For MTT assay, U87-MG cells and GBM2 CSCs were plated in 96-well dishes, at 2 × 10^3^ cells/well, whereas C6 cells were plated at 1 × 10^3^ cells/well; after 24 h, cells were treated with the indicated concentrations of RGWE and rutin. Cell staining was performed at the indicated times, using 5 mg/mL Thiazolyl Blue tetrazolium bromide (MTT) (Invitrogen-Life Technologies, Eugene, OR, USA) in PBS. Cells were incubated for 3 h at 37 °C, at a 10% final concentration, followed by the addition of 100 μL of DMSO. Then, optical densities were measured at 495 nm by a Microplate Reader (Synergy HT, Bio-Tek, VT, USA).

Cell viability rate was monitored for 72 h in the case of GBM2 CSCs and for 24 h in the case of glioma cell lines, and expressed as a percentage by dividing the mean absorbance of the treated groups to the one of the control groups, as follows:

Cell viability rate (%) = (A_treated group_/A_control group_) × 100

### 4.10. RNA Extraction

To evaluate the expression of specific genes in the glioblastoma cell line, in the presence and/or absence of RGWE, RNA extraction was conducted. Briefly, U87-MG cells were plated at 3 × 10^5^ cells/well in 6 multi-wells plates, and after 24 h cells were treated with specific effectors at specific concentrations. RNA extraction was performed by TRIreagent (ThermoFisher) according to manufacturer’s instructions. Total RNA concentration was measured by a Nanodrop 2000c (ThermoScientific, Milano, Italy).

### 4.11. cDNA Retrotranscription

cDNA was synthesized from 1 µg of total RNA using a high-capacity cDNA reverse transcription kit (Applied Biosystem, Waltham, MA, USA) and random hexamer oligonucleotides according to the manufacturer’s instructions and using the following thermal cycle protocol (Table 1).

### 4.12. Real-Time Polymerase Chain Reaction

cDNA was then used for real-time PCR.

The forward (f) and reverse (r) primers (5′–3′) used were as follows:

- Human VEGF f ATCTTCAAGCCATCCTGTGTGC and r CAAGGCCCACAGGGATTTTC;
- Human GAPDH f ATGACATCAAGAAGGTGGTG and r CATACCAGGAAATGAGCTTG.

Amplification reactions were run in triplicate in MicroAmp 96-well plates (Applied Biosystems) containing 2 μL cDNA (100 ng), a primer mix at a final primer concentration of 200 nM each, and 10 μL of 2× Syber Green Master Mix (Applied Biosystem) in a final volume of 20 μL. Amplifications were carried out in a StepOne Plus (from Applied Biosystems). Reactions started with an initial step of 95 °C for 15 min to activate Taq Polymerase. The reaction proceeded with 40 cycles of 95 °C for 15 s, 60 °C for 1 min, and 72 °C for 20 s for gene amplification. A final dissociation step was always performed to obtain the melting curves (thermal profile) of the amplicons obtained in the reactions. mRNA levels were expressed as 2^−ΔCt^ using the GAPDH mRNA level as the internal standard.

### 4.13. Statistical Analysis

Data were expressed as mean ± standard deviation (SD) of three separate experiments, indicated by error bars. A Student *t* test was conducted to analyze differences between data sets, indicated in the figures with *p*-values ≤0.05 (*), ≤0.005 (**), or ≤ 0.001 (***).

## Figures and Tables

**Table 1:** Protocol used for cDNA retrotranscription.

	Step 1	Step 2	Step 3	Step 4
Temperature	25 °C	37 °C	85 °C	4 °C
Time	10 min	120 min	5 min	ꟹ

## Data Availability

The data used to support the current study are available from the corresponding author upon reasonable request.

## Supplementary Materials

The following supporting information can be downloaded at: https://www.mdpi.com/article/10.3390/ijms252111789/s1.
