# Supplementary material for: Ruta graveolens, but Not Rutin, Inhibits Survival, Migration, Invasion, and Vasculogenic Mimicry of Glioblastoma Cells
Source: Int J Mol Sci. 2024 Nov 2;25(21):11789. doi: 10.3390/ijms252111789 (PMC11546663; doi:10.3390/ijms252111789)
Supplement: Supplementary file 1 [file ijms-25-11789-s001.zip › ijms-3233352-supplementary.pdf]

Supplementary materials

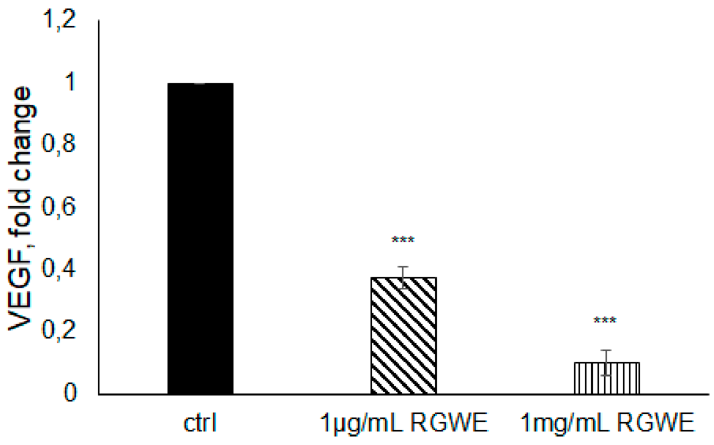

Supplementary Figure 1. RGWE modulates mRNA expression levels of VEGF. Representative real time PCR analysis of the expression of VEGF mRNA in ctrl group and treated groups with increasing doses of RGWE (1 µg/mL and 1 mg/mL). As shown, RGWE reduced VEGF mRNA expression levels. *** p-value < 0.001.
